# Supplementary figures and images for: The yield of community-based tuberculosis and HIV among key populations in hotspot settings of Ethiopia: A cross-sectional implementation study
Source: PLoS One. 2020 May 29;15(5):e0233730. doi: 10.1371/journal.pone.0233730 (PMC7259557; doi:10.1371/journal.pone.0233730)

**Supporting information**

**S1 Figure: Study settings, the selected hotspot towns in Ethiopia.**


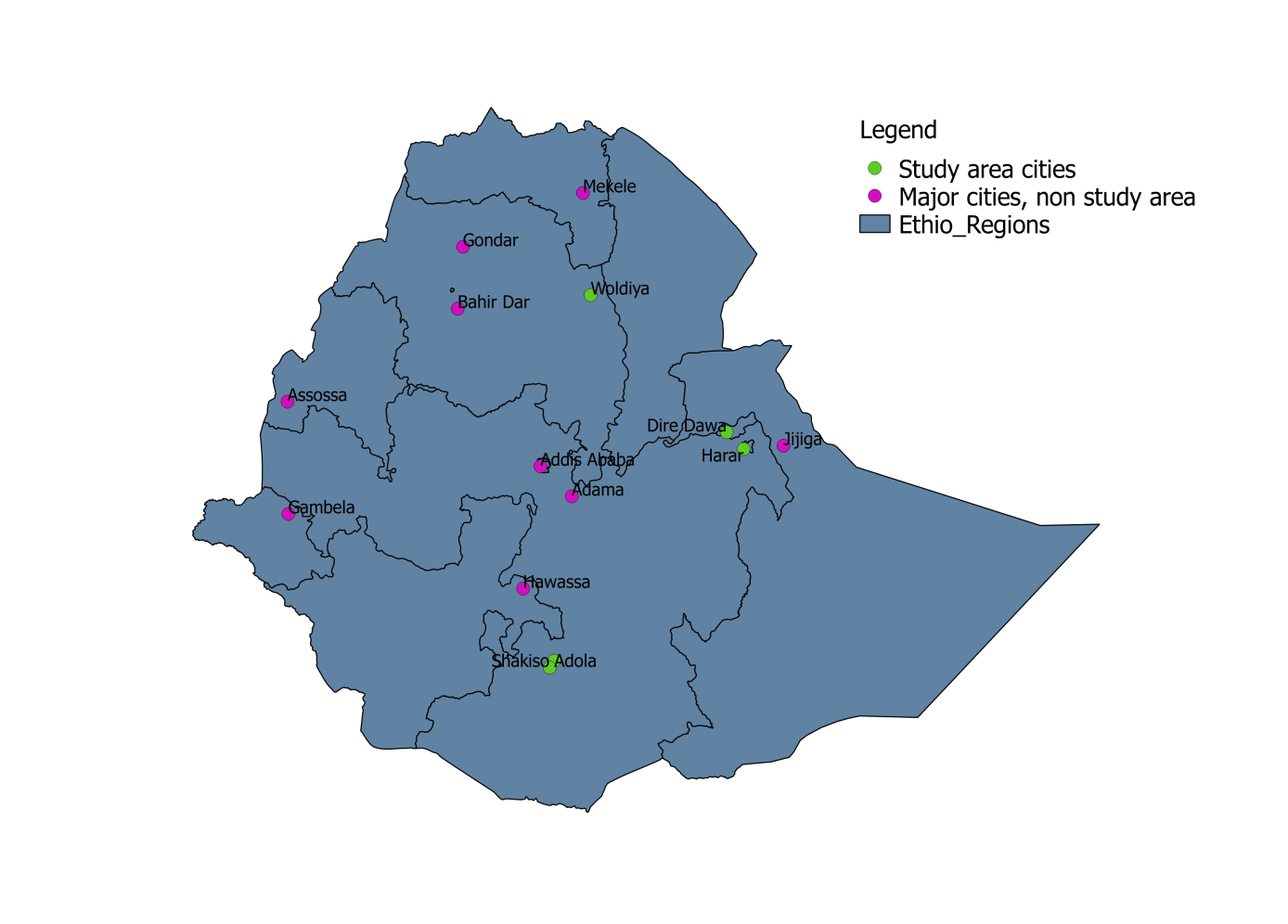

Supplement: S1 Fig — The cities in red colors are bigger cities in Ethiopia and are also the capital cities of the regions; they had less than 10% TB/HIV co-infection. The area with green-yellow color spot are the study towns assigned as hotspot settings for the TB and HIV, with TB/HIV co-infection of at least 10%. (DOCX) [file pone.0233730.s001.docx]
