# Supplementary material for: The yield of community-based tuberculosis and HIV among key populations in hotspot settings of Ethiopia: A cross-sectional implementation study
Source: PLoS One. 2020 May 29;15(5):e0233730. doi: 10.1371/journal.pone.0233730 (PMC7259557; doi:10.1371/journal.pone.0233730)
Supplement: S1 Table — Age is categorized based on the median of the overall population. In the marital status, divorced and widowed were merged to show a key population without a partner. The educational category was based on grading of the Ethiopian education system. Numbers in the bracket are to show the percentage. (DOCX) [file pone.0233730.s002.docx]

**S1 Table: The number and proportion of key populations based on their socio-demographic characteristics in the five towns of Ethiopia, August 2017-January 2018**

| **Variables** | **Category** | **Type of key population** | | | | | | |
| --- | --- | --- | --- | --- | --- | --- | --- | --- |
|  |  | **IMW, # (%) N=237** | **Prison inmates, # (%), N=1112** | **FSW, # (%), N=221** | **Residents in the missionary charity, # (%) N=79** | **Homeless, #(%) N=14** | **HCWs # (%) N=113** | **IDP, # (%) N=102** |
| Age | *1. <28 years* | 100 (44.8) | 532 (47.9) | 96 (43.4) | 24 (30.4) | 7 (50) | 71 (62.8) | 55 (53.9) |
|  | *2. >=28 years* | 123 (55.2) | 579 (52.1) | 125 (56.6) | 55 (69.6) | 7 (50) | 42 (37.2) | 47 (46.1) |
| Sex | *1. Female* | 74 (31.2) | 199 (17.9) | 221 (100) | 32 (40.5) | 7 (50) | 29 (25.7) | 46 (45.1) |
|  | *2. Male* | 163 (68.8) | 913 (82.1) | 0 (0) | 47 (59.5) | 7 (50) | 84 (74.3) | 56 (54.9) |
| Marital status | *1. Married/has parter* | 94 (39.7) | 624 (56.1) | 100 (45.3) | 39 (49.4) | 4 (28.6) | 20 (17.7) | 30 (29.4) |
|  | *2. Single/never married* | 75 (31.7) | 349 (31.4) | 98 (44.2) | 21 (26.6) | 4 (28.6) | 60 (53.1) | 51 (50) |
|  | *3. Divorced/separated* | 21 (8.9) | 137 (12.3) | 14 (6.3) | 15 (19) | 4 (28.6) | 33 (29.2) | 21 (20.6) |
|  | *4. Widowed* | 47 (19.8) | 2 (0.2) | 9 (4.0) | 4 (5.1) | 2 (14.2) | 0 | 0 |
| Educational status | *1. 1-6 grade* | 46 (19.4) | 604 (54.3) | 64 (29) | 47 (59.5) | 8 (57.1) | 49 (43.4) | 37 (36.3) |
|  | *2. 7-8 grade* | 163 (68.7) | 391 (35.2) | 80 (36.2) | 23 (29.1) | 2 (14.3) | 13 (11.5) | 43 (42.2) |
|  | *3. 9-12 grade* | 23 (9.7) | 106 (9.5) | 21 (9.5) | 7 (8.9) | 4 (28.6) | 7 (6.2) | 16 (15.7) |
|  | *4. >=12 above* | 5 (2.1) | 11 (1) | 56 (25.3) | 2(2.6) | 0 | 44 (38.9) | 6 (5.6) |
