# Supplementary material for: The yield of community-based tuberculosis and HIV among key populations in hotspot settings of Ethiopia: A cross-sectional implementation study
Source: PLoS One. 2020 May 29;15(5):e0233730. doi: 10.1371/journal.pone.0233730 (PMC7259557; doi:10.1371/journal.pone.0233730)
Supplement: S2 Table — HIV counseling and request for test was offered for 1293 key populations. Testing was offered for 1111 of them. 182 refused testing, and the other 585 HIV counselling and testing was not undertaken due to the shortage of HIV testing kits. Therefore, the respective TB screened 182 and 582 key populations were not tested for HIV due to refusal and shortage of HIV screening test kits. (DOCX) [file pone.0233730.s003.docx]

**S2 Table 2: The status of HIV, TB/HIV, and linkage to ART services in the selected five hotspot towns in Ethiopia, August 2017- January 2018**

| **Variable** | **Frequency** | **Percent** |
| --- | --- | --- |
| **If tested for HIV, test result** | | |
| HIV positive | 67 | 5.18 |
| HIV negative | 1,044 | 80.74 |
| Refused the test | 182 | 14.08 |
| Total counseled for HIV testing | 1,293 | 100 |
| **Linkage and ART status of HIV infected** | | |
| On HIV care (ART or pre-ART) | 49 | 73.1 |
| Just linked to HIV care | 14 | 20.9 |
| Not linked to HIV | 4 | 6.0 |
| Total | 67 | 100 |
| **TB disease among HIV infected** |  |  |
| TB case | 21 | 31.3 |
| No TB case | 46 | 68.7 |
| Total PLHIV | 67 | 100 |
| **HIV status among TB cases** |  |  |
| HIV positive | 21 | 36.8 |
| HIV negative | 20 | 35.1 |
| Refused test | 16 | 28.1 |
| Total TB cases approached for HIV testing | 57 | 100 |
