# Supplementary material for: The yield of community-based tuberculosis and HIV among key populations in hotspot settings of Ethiopia: A cross-sectional implementation study
Source: PLoS One. 2020 May 29;15(5):e0233730. doi: 10.1371/journal.pone.0233730 (PMC7259557; doi:10.1371/journal.pone.0233730)
Supplement: Attachment — Submitted filename: PONE-D-20-01766.docx [file pone.0233730.s004.docx]

**Comments to the authors:**

The cross-sectional study by Dememew ZG et al. deals with the prevalence of TB and HIV in few hot-spots of Ethiopia. Ethiopia is among the top 10 high TB/HIV coinfection prevalence state. Studies are needed which identify TB or TB/HIV coinfection hot spots and also suggest measures to curb these deadly human menaces. Within the identified hot spots, they selected vulnerable population educated them about the importance of TB screening and HIV testing prior to the recruitment of the study. Although, the incidence of TB has dropped in most regions of the world including Ethiopia. However, TB with HIV coinfection is largely prevalent in vulnerable or socially excluded populations and high-risk settings. The studies are required which keep track vulnerable populations and putting them at forefront of interventions to effectively diagnose all the TB cases as well as TB/HIV coinfection. In the current study the authors detected more HIV and TB cases among vulnerable and key populations in the hotspot settings compared to the general population. This study also suggests the importance of community-based collaborations of TB and HIV programmes. The study is well designed, executed and conclusions drawn are well supported by results. The manuscript is very well written and the information provided will greatly help in the management and diagnosis of TB as well as TB/HIV coinfection cases in hot spots of Ethiopia. I have no specific reservations and suggestions for the authors. The manuscript could be considered for publication in PLOS one.
