# Supplementary material for: The yield of community-based tuberculosis and HIV among key populations in hotspot settings of Ethiopia: A cross-sectional implementation study
Source: PLoS One. 2020 May 29;15(5):e0233730. doi: 10.1371/journal.pone.0233730 (PMC7259557; doi:10.1371/journal.pone.0233730)
Supplement: Attachment — Submitted filename: Response to Reviewers.docx [file pone.0233730.s005.docx]

Date: *April 20, 2020*

From: *Zewdu Gashu Dememew (MD, MPH, PhD)*

To: *PLOS ONE*

Issue*:* ***'Point-by-point Response to Reviewers’ comments***

Dear Editors,

It is to be remembered that the article entitled,” *The yield of community-based enhanced tuberculosis and Human Immuno-deficiency virus case finding among key populations in selected hotspot settings of Ethiopia: a cross-sectional implementation study”* with the manuscript Number of PONE-D-20-01766 was submitted to your journal and was reviewed by two reviewers.

Accordingly, we have tried to address the comments by the reviewers and the editor as well. We have found the comments very important and constructive. Therefore, we have looked into the comments and enriched the article again. We have responded to the comments point-by-point in the table attached below where the first column (**in bold)** is the comments by the reviewer and the second one is the responses (***italic****).*

We have also tried to shape the manuscript per the guideline of the PLOS ONE. Once again, we want to ensure you that this article is not submitted to other journal and all the authors agreed the re-submission to your journal. Eventually, we request your organization to review our response and revise manuscript for the second round and consider for publication.

The authors are always ready to give a prompt respond for any comment from the reviewers or the editor of the PLOS ONE.

Sincerely yours

Zewdu Gashu Dememew

Editor: COMMENTS TO AUTHORS PONE-D-20-01766

| **Editor comment/suggestion** | Response |
| --- | --- |
| **Please ensure that your manuscript meets PLOS ONE's style requirements, including those for file naming. The PLOS ONE style templates can be found at http://www.plosone.org/attachments/PLOSOne_formatting_sample_main_body.pdf and http://www.plosone.org/attachments/PLOSOne_formatting_sample_title_authors_affiliations.pdf** | *The required style, the file naming etc… are amended per the PLOS One requirement* |
| **2.    Please provide additional information regarding the considerations made for the prisoners included in this study. For instance, please discuss whether participants were able to opt out of the study and whether individuals who did not participate receive the same treatment offered to participants.** | *In the Ethical consideration section”* *All the key and vulnerable population were informed that it is their full right to exit from the study if they are not willing. However, all were getting TB screening, evaluation and treatment services irrespective of their willingness or refusal to participate in the study.” Lines:223-226 in the Manuscript* |
| **3.    Please address the following:**  **- Please ensure you have thoroughly discussed any potential limitations of this study within the Discussion section, for example, the skewed nature of the dataset towards males.**  **- Please refer to any sample size calculations performed prior to participant recruitment. If these were not performed please justify the reasons. Please refer to our statistical reporting guidelines for assistance (**[**https://journals.plos.org/plosone/s/submission-guidelines.#loc-statistical-reporting**](https://journals.plos.org/plosone/s/submission-guidelines.#loc-statistical-reporting)**).**  **- Please modify the title to ensure that it is meeting PLOS’ guidelines (https://journals.plos.org/plosone/s/submission-guidelines#loc-title). In particular, the title should be "specific, descriptive, concise, and comprehensible to readers outside the field". In this case, we feel that the title is not concise, for example HIV could be used instead of "Human Immuno-deficiency virus".** | *In the limitation part; ‘**The data is skewed towards male for there were a lot of male HCWs, prison-in-mates and IDP, possibly posing the challenge of generalization to a general population to similar settings’’ Lines:402-403 in the Manuscript*  *As this is an implementation study sample size calculation was not determined during the initial phase of the study*  *Thank you for the comment and the title is modified as, “The yield of community-based tuberculosis and HIV among key populations in hotspot settings of Ethiopia: a cross-sectional implementation study”* |
| **4.    Thank you for your ethics statement : "The ethical review committee (ERC) of the respective regions of the study towns approved the study protocol."**  **Please amend your current ethics statement to include the full name of the ethics committee/institutional review board(s) that approved your specific study.**  **Once you have amended this/these statement(s) in the Methods section of the manuscript, please add the same text to the “Ethics Statement” field of the submission form (via “Edit Submission”).**  **For additional information about PLOS ONE ethical requirements for human subjects research, please refer to http://journals.plos.org/plosone/s/submission-guidelines#loc-human-subjects-research.** | *This is right and addressed as, “The ethical review committee (ERC) of the respective regions of the study towns such as Amhara and Oromia regions, and Harari and Diredawa cities” Lines: 215-217.*  *The same ethical statement is added to the field of submission.* |
| **Please amend the manuscript submission data (via Edit Submission) to include author D. Gemechu, D. Bekele, G. Yismawu, K.Melkieneh, B. Reshu, P.G. Suarez.**    **Please amend your authorship list in your manuscript file to include author Daniel G Datiko.** | *It is true these were not considered initially and now all become part of the submission* |
| **Please include your tables as part of your main manuscript and remove the individual files. Please note that supplementary tables (should remain/ be uploaded) as separate "supporting information" files**  **We note that Figure S1 in your submission contain map images which may be copyrighted. All PLOS content is published under the Creative Commons Attribution License (CC BY 4.0), which means that the manuscript, images, and Supporting Information files will be freely available online, and any third party is permitted to access, download, copy, distribute, and use these materials in any way, even commercially, with proper attribution. For these reasons, we cannot publish previously copyrighted maps or satellite images created using proprietary data, such as Google software (Google Maps, Street View, and Earth). For more information, see our copyright guidelines: http://journals.plos.org/plosone/s/licenses-and-copyright.**    **Please include captions for your Supporting Information files at the end of your manuscript, and update any in-text citations to match accordingly. Please see our Supporting Information guidelines for more information: http://journals.plos.org/plosone/s/supporting-information.** | *This is done as suggested. Thank you dear editor.*  *This is our own and original map. It has not been used anywhere so far, and prepared by one of the authors (Nebiyu Hiruy, M/E expert). The figure or the map is not taken from any website. If it is needed or requested, we can provide the original data set (coordinates of each cities in Ethiopia) from where the map is sketched.*  *This is prepared.* |

REVIEWER # 1: Review of manuscript PONE-D-20-01766

| **Reviewer comment** | Authors Response and amendments/revision |
| --- | --- |
| **1. The manuscript requires language editing all through the manuscript including technical terms eg. line 160 (illness is out of lung tissue: meaning extrapulmonary TB) Authors are requested to take professional help in undertaking this** | *Yes, this is true. And the second-round extensive language edit was undertaken by a professional language editor and then acknowledged; “…Barbara K. Timmons for editing the manuscript” Line: 423-424* |
| **2. Line 97: Please describe who are the TB focal persons** | *This is described as,” HCWs that coordinate comprehensive TB and TB/HIV activities, “Line: 99* |
| **3. Line 120 : It would be helpful to know what the morbidity was at the time of data collection and which study population they represented. This could be represented in the Flow Chart which incidentally needs to be enumerated as Fig.**  **subpopulations in the cohort. The findings need to be analyzed and represented in much more depth.** | *This is a sound suggestion and described as,”… sick of other illnesses other than TB or TB like symptoms such as malaria and other acute febrile illnesses (40), and refused to participate (11)”. In each key population the number of refusal and the sick were written. E.g. Prisoners: 1125 (9 sick, 4 refused) Missionary residents: 87 (8 sick) HCWs: 123 (6 refused, 4 sick); FSW:225 (1 refused,3 sick) IMW:245 (8 sick) IDP:105 (3 sick) Homeless: 19 (5 sick). See* ***Figure 1.***  *This is also described in the caption as, “**Most of the refusal were from HCW and FSW for they were busy, and from prison inmates. The malaria illness and other acute febrile illness made the other key population difficult to participate in the study.” Lines: 159-161* |
| **4. Whilst acceptance of TB and HIV screening is mentioned in the text, it would be helpful to know whether consent for both TB and HIV screenings was undertaken simultaneously or separately and were there instances where TB screening was acceptable but not HIV or vice versa** | *There was a separate consent request for TB screening and HIV screening and described under ethical consideration. “**Even though a separate consent requested for TB screening and HIV screening, it was asked one after the other; first for TB screening and then for HIV testing” Lines: 221-223*  *Accordingly,’* *HIV counseling and request for test was offered for 1293 key populations. Testing was offered for 1111. 182 refused testing and for the other 585 HIV counselling and testing was not undertaken due to the shortage of HIV testing kits. “Therefore, the respective TB screened 182 and 582 key populations were not tested for HIV due to refusal and shortage of HIV screening test kits.” Described in S2Table caption Lines: 626-630* |
| **5. In a process driven paper such as this, it would be desirable to know the process in qualitative terms viz. where were the screenings undertaken, how long did it take to do the screenings and challenges during screening.** | *The place of screening is at the work placed where the key population were recruited;” when the HEWs and TB focal persons visited the FSWs in the hotels, IMWs at the workplace, IDPs in the refugee centers and homeless in the street, they registered these key population on excel sheet (sampling frame).” Lines: 132-135*  *The time spent is now described as ;”* *For a single key population, the time spent for recruitment, TB screening, HIV testing and counselling, and recording all the outcome of screening and evaluation for TB ranged from 15-20 minutes.” 173-176*  *The challenges during the screening was mentioned as one of the limitations as,”* *The living situation of the homeless and the working environment of the FSW challenged the sampling and data gathering’’. Lines: 407-408* |
| **6. Line 160-163 is confusing. Do the authors imply that follow up of the confirmed TB patient was necessitated for a retrospective classification. All the more reason for the work -low to be accurately described.** | *_We have reported active TB cases detected during the study period. This was recorded after screening and evaluating (clinical and lab investigation) the key population for TB. For instance, soon after the key population is screen positive for TB, s/he was sent/referred to nearby health facility for TB evaluating. Then, the outcome was recorded and reported and became part of this study._*  *_However, these lines are to define the standard classification/categories of TB per the national guideline. Lines: 177-184_* |
| **7. The lack of uniformity in confirming TB diagnosis needs to be explained and the implications in computing prevalence if any**  **(lines 216-218)** | *The defined category of TB and standard ways of TB diagnosis (Lines 177-184) was described to have a uniform ways of confirming TB diagnosis. Hence, there is no need to explain these here.* |
| **8. An interesting but surprising finding is the minimal amount of TB detected in migrants. Were these recent or long-term migrants and what was the disease status in the areas they migrated from.** | *This was discussed and justified. These were recent migrant that arrived almost 4-6 weeks prior to data collection. These were internally displaced people due to communal conflicts and most of them were recent arrival and healthy individual during the data collection. Lines:117-118, and 390-392* |
| **9. Again a similar scenario in homeless persons generally considered to be one of the most vulnerable. Where did these persons live: on the streets or were they institutionalized/ congregated in any way. Descriptions are necessary to understand the observations** | *These were living on the street; ,” … TB focal persons deployed homeless individuals in the street”. Lines: 11. The justification was also described in discussion section. “The homeless were identified as one of the vulnerable and key populations, although they were few, with no TB and HIV cases identified” line:383-389* |
| **10. The observations do not have break up of findings as per age and gender though the information is partially provided in the tables with minimal stratification. Difficult to vizualize whether the high prevalence figures computed define certain subpopulations in the cohort. The findings need to be analyzed and represented in much more depth.** | *This is true, and the* ***supplement table 1*** *described the age and gender category of the key population. However, detailed age and gender-based TB prevalence for each key population was not entertained for a general skewedness of data towards male gender. For instance, all FSW were females and more than three-fourth of HCWs and Prison inmates were males.* |
| **11. The discussion provides valuable insights and touches upon the risk to to HCW, the need to converge services for TB HIV (and NCDs also as in other parts of the world) and the overall lack of infection control. The low prevalence of TB in IDP is well explained. Therefore it would benefit the manuscript in the Results section to include the temporal data of the movement of the displaced persons.** | *Displaced people were from the neighboring region (Somali) and district with similar TB case detection. It only takes 1-2 hours of movement/travel to arrive at the Harar from Jijgiga of Somali region. Hence, “*temporal data of the movement of the displaced persons. *“was very difficult to describe.* |
| **12. The last sentence needs to be better formatted.** | *This is formatted as suggested.* |
| **13. The message of comprehensive care through service convergence is one that is valuable in many settings and should not be ignored.** | *Thank you. That is acceptable and addressed in the conclusions as ,” community-based collaboration of TB and HIV programs … ” Line:419-420 and “These suggest a community and health facility based integrated … case finding approaches for TB and HIV...” Line: 27-28* |
